# Quality of life of breast and cervical cancer survivors

**DOI:** 10.1186/s12905-017-0387-x

**Published:** 2017-04-12

**Authors:** Huei-Ying Huang, Wen-Chen Tsai, Wen-Yu Chou, Yao-Ching Hung, Liang-Chih Liu, Kuo-Feng Huang, Wen-Ching Wang, Kam-Wing Leung, Ruey-Kuen Hsieh, Pei-Tseng Kung

**Affiliations:** 1 Department of Anesthesia, Chung Shan Medical University Hospital, Taichung, Taiwan, Republic of China; 2 Department of Health Service Administration, China Medical University, Taichung, Taiwan, Republic of China; 3 Department of Gynecologic Oncology, China Medical University Hospital, Taichung, Taiwan, Republic of China; 4 Department of Breast Surgery, China Medical University Hospital, Taichung, Taiwan, Republic of China; 5 Cancer Center, Chi Mei Hospital, Tainan, Taiwan, Republic of China; 6 Department of General surgery, Chi Mei Hospital, Tainan, Taiwan, Republic of China; 7 Dental Department, Yuan’s General Hospital, Kaohsiung, Taiwan, Republic of China; 8 Division of Hematology and Oncology, Department of Internal Medicine, Mackay Memorial Hospital, Taipei, Taiwan, Republic of China; 9 Department of Healthcare Administration, Asia University, 500, Lioufeng Road, Wufeng, Taichung, 413 Taiwan, Republic of China; 10 Department of Medical Research, China Medical University Hospital, China Medical University, Taichung, Taiwan, Republic of China

**Keywords:** Breast cancer, Cervical cancer, Cancer survivors, Quality of life

## Abstract

**Background:**

Breast and cervical cancer are the most common cancers affecting women. The symptom distresses experienced by cancer survivors are critical factors influencing their quality of life (QOL). This study investigated the QOL of breast and cervical cancer survivors, their physical, psychological and social conditions.

**Methods:**

The participants were older than 20 years, had been diagnosed with breast or cervical cancer for more than 2 years, and had completed their cancer treatment. The survey incorporated the QOL questionnaires developed by the European Organization of Research and Treatment for Cancer and a self-designed questionnaire.

**Results:**

The mean age at diagnosis was 48.89 ± 8.53 years for the breast cancer survivors and 49.00 ± 10.30 years for the cervical cancer survivors. The corresponding QOL scores were 75.33 ± 20.25 and 75.56 ± 17.93. The factors influencing QOL of breast cancer survivors were household income, number of comorbidities, stage of cancer, type of cancer treatment and duration of illness, whereas the factor related to QOL of cervical cancer survivors was only household income.

**Conclusions:**

The QOL of the two groups was similar. Healthcare providers should demonstrate greater concern toward breast and cervical cancer survivors.

## Background

The International Agency for Research on Cancer reported that the number of new cancer cases worldwide was 14.1 million in 2012 [1]. Breast and cervical cancer are common cancers among women, affecting approximately 1.67 million and 530,000 people each year worldwide, respectively. In 2012, 522,000 and 270,000 women died of breast and cervical cancer, respectively, accounting for the highest and fourth-highest mortality rates among women [2].

According to the Ministry of Health and Welfare, Taiwan, cancer has been the leading cause of death in Taiwan for 32 consecutive years, accounting for 29% of all deaths in 2013, and the standardized mortality rate was 130.4 per 100,000 persons [3]. As indicated by the Health Promotion Administration of Taiwan, breast and cervical cancer are the most hazardous illnesses affecting women’s health; if detected early, the 5-year survival rates for women with breast and cervical cancer are 88.7–97.5% and 88.8–96.8%, respectively [4].

The World Health Organization defined quality of life (QOL) as involving a person’s physical health, psychological state, degree of independence, social relationships, personal beliefs and environment [5]. Female cancer survivors are faced with physical, psychological and social distress in addition to fatigue, irritability, memory loss, decreased energy level, recurring pain and decreased QOL [6–9]. The symptom distress experienced by cancer survivors is a critical factor influencing their QOL [10, 11]. Following a cancer diagnosis, patients with breast and cervical cancer must undergo a series of long-term treatments and follow-up examinations to prevent recurrence and improve survival [12]. In addition to symptom distress resulting from cancer treatment and physical discomfort caused by the illness, other factors, such as body image impairment, psychological fear, anxiety and low self-esteem, can affect the QOL of patients [8, 11, 13]. After undergoing surgery and other forms of treatment, patients with breast cancer frequently experience various types of discomfort, including stomach upset, a lack of appetite, nausea, vomiting, fatigue, arm pain and difficulty breathing, all of which introduce many inconveniences into their lives and greatly influence their QOL [10, 14, 15]. In the early treatment of cervical cancer, the most common types of discomfort and the primary factors influencing the long-term QOL of patients are constipation, lower extremity edema, urinary incontinence, hot flashes, diarrhea and increased vaginal discharge [16].

Although numerous studies have separately measured the QOL of cancer patients, few studies have explored the QOL of breast and cervical cancer survivors. Because breast and cervical cancer are among the top ten cancers in terms of incidence in Taiwan and are both cancers affecting women, we simultaneously addressed these two types of cancer in the present study. We investigated the QOL of breast and cervical cancer survivors, as well as their physical, psychological and social conditions. In addition, we explored whether patients characteristics, health status, household income, health behaviors, disease severity, medical care provider characteristics, treatment methods and illness duration were associated with the patients’ QOL.

## Methods

### Participants

This adopted a cross-sectional design. In accordance with Klug et al., we defined cancer survivors as patients who survived for more than 2 years following a cancer diagnosis [17]. Breast and cervical cancer survivors were recruited according to the following inclusion and exclusion criteria. Inclusion criteria: patients who were older than 20 years, had been diagnosed with breast or cervical cancer for >2 years, and had completed their cancer treatment (i.e., were currently receiving only regular follow-ups). Exclusion criteria: patients who were currently receiving cancer treatment (excluding those with breast cancer who were currently undergoing hormone therapy [HT]), who had terminal cancer, whose cancer stage was unknown, who had multiple types of cancer, who had carcinoma in situ, and who were men with breast cancer.

### Sampling and data collection

Patient consent for this study was received from the cooperating hospitals and approved by the institutional review boards (IRB No. CMU-REC-101-013). According to the proportion of patients at the cooperating hospitals, as indicated by the Taiwan Ministry of Health and Welfare, we calculated the number of cases that should be collected from five medical centers and three regional hospitals in Northern, Central and Southern Taiwan. Subsequently, interviewer training was conducted and doctors, nurses, and case managers at the collaborating hospitals were entrusted with selecting and recruiting respondents who met the inclusion criteria. After patient consent was obtained, the questionnaire surveys were distributed.

### Questionnaire design and content

The questionnaire used in this study incorporated QOL questionnaires (QLQs) developed by the European Organization of Research and Treatment for Cancer (EORTC) as well as a self-designed questionnaire. The questionnaire was designed to elucidate the QOL of cancer survivors.

To assess the QOL of the participants, we acquired permission to use the Chinese version of the EORTC questionnaires, including the core QLQ-C30 and QLQ supplementary modules [16, 18–21] for breast cancer QLQ (QLQ-BR23) and cervical cancer QLQ (QLQ-CX24). The QLQ-C30 contains 30 questions, comprising five groups of questions on functioning, namely physical functioning (five questions), role functioning (two questions), emotional functioning (four questions), cognitive functioning (two questions) and social functioning (two questions); two questions on global health status and QOL; and 12 questions on the symptoms or problems frequently experienced by patients with cancer, namely fatigue (three questions), pain (two questions), nausea and vomiting (two questions), difficulty breathing (one question), insomnia (one question), lack of appetite (one question), constipation (one question) and financial difficulty (one question).

The QLQ-BR23 contains eight questions, comprising four groups of questions on functioning, namely body image (four questions), sexual functioning (two questions), sexual enjoyment (one question) and future perspective (one question); and 15 questions on the symptoms or problems frequently experienced by patients with breast cancer, namely side effects from treatment (seven questions), symptoms related to the breasts (four questions), symptoms related to the arms and shoulders (three questions), and hair loss (one question).

The QLQ-CX24 contains nine questions, comprising four groups of questions on functioning, namely body image (three questions), sexual activity (one question), sexual enjoyment (one question) and sexual/vaginal functioning (four questions); and 15 questions on the symptoms or problems frequently experienced by patients with cervical cancer, namely general symptoms (11 questions), lymphatic symptoms (one question), peripheral neuropathy (one question), menopausal symptoms (one question) and sexual anxiety (one question).

The EORTC questionnaire has been employed in previous studies to clarify the QOL of the cancer survivors [21–24]. A high score on the global health status (i.e., overall QOL score) and functioning dimension (0–100) represents a high level of QOL and functioning, whereas a high score on a symptom dimension (0–100) indicates a high level of (or severe) symptoms. The EORTC questionnaire is widely known to demonstrate acceptable validity [16, 19, 24, 25]. In the present study, the reliability Cronbach’α coefficients of the EORTC QLQ-30 and QLQ-BR23 for breast cancer survivors (*n* = 252) were 0.93 and 0.88, respectively and those of the EORTC QLQ-30 and QLQ-CX24 for cervical cancer survivors (*n* = 75) were 0.91 and 0.81, respectively.

### Self-designed questionnaire and its validity

On the basis of related literature and data, the content of the self-designed questionnaire was divided into two major parts: one part was completed by the respondents and the other part was completed by the healthcare professionals after reviewing the patients’ medical records. The part completed by the respondents incorporated items from four major categories: health condition (e.g., disease history and medical history), lifestyle (e.g., cigarette smoking, alcohol consumption and exercise), (4) demographic information (e.g., sex, educational attainment, marital status, living conditions and household income). The part completed by the healthcare professionals comprised items on the therapeutic situation of the patients (e.g., date of diagnosis, stage of cancer, methods of treatment and whether the cancer recurred or metastasized). The content validity of the self-designed questionnaire was assessed by seven experts, including specialists, case managers, cancer registrars and scholars, who were invited to assess the content of the questionnaire for survivors of the two types of cancer. The questionnaire was revised according to feedback from the experts, with ambiguous questions reworded for clarity to ensure the integrity of the instrument. Subsequently, the content validity index (CVI) was employed to evaluate the revised questionnaire according to the mean CVI; in general, a mean CVI of >0.8 indicates adequate validity [26, 27]. The mean CVIs derived from the self-designed questionnaire for breast cancer and cervical cancer survivors were 0.98 and 0.97, respectively, indicating a high level of validity. The self-designed questionnaire used in our study contained items on personal information and disease information; these items were unrelated to the conception of any specific dimension and were thus unsuitable for any reliability analysis.

### Statistical analysis

The frequencies, percentages and means of the following demographic characteristics were statistically analyzed: personal characteristics (sex, age and educational attainment), socioeconomic status (household income), social support (marital status and living conditions), health conditions (comorbidities), specifics pertaining to cancer treatment (cancer stage, recurrence or metastasis, treatment method, illness duration and medical institution level), lifestyle (e.g., cigarette smoking, alcohol consumption and exercise), participants’ apprehension regarding the recurrence or metastasis of cancer, and the various dimensions of QOL.

A t test and one-way analysis of variance (ANOVA) were conducted on the following characteristics: personal characteristics (sex, age and educational attainment), household income, social support (marital status), health conditions (comorbidities), specifics pertaining to cancer treatment (cancer stage, recurrence or metastasis, treatment method, illness duration and medical institution level), and global health status. In addition, the participants who received treatment at the same hospitals may exhibit homogeneity, and cancer treatments offered in a single hospital are similar in nature; therefore, the cluster effect of participants from the same hospital was considered. Medical institution was employed as a repeated measure variable in the multiple regression model analysis using generalized estimating equations, in which the global health status was taken as the dependent variable, and the patients’ personal characteristics, socioeconomic status, social support, health condition and specifics pertaining to cancer treatment were taken as independent variables. Among the independent variables, in addition to the variables that achieved *p* < 0.25 in the bivariate analysis, the two variables “cancer stage” and “cancer recurrence or metastasis” were used as control variables in analyzing and exploring the relevant factors influencing the QOL of the cancer survivors.

## Results

### Basic characteristics

A total of 327 valid questionnaires were returned by the breast cancer survivors (*n* = 252) and cervical cancer survivors (*n* = 75). In Table 1, the mean age at diagnosis was 54.48 ± 8.33 years for the breast cancer survivors and 55.26 ± 10.50 years for the cervical cancer survivors, showing that these two groups were of similar age. Participants who had graduated from high school or vocational school accounted for the highest percentage of all participants (breast cancer, 42.06%; cervical cancer, 26.67%), and most participants were married (breast cancer, 75%; cervical cancer, 70.7%). The monthly household income among the breast cancer patients was relatively even, with the largest group (50.80%) earning > US$2,001. Among the cervical cancer patients, the largest group (36.00%) earned < US$1,000.

**Table 1:** Characteristics and therapeutic situations of the participants

Variable	Breast cancer (*N* = 252)		Cervical cancer (*N* = 75)	
	*n*	%	*n*	%
Age (years)	Mean:54.48 ± 8.33		Mean:55.26 ± 10.50	
≦44	23	9.13	11	14.67
44–54	107	42.46	27	36.00
55–64	97	38.49	23	30.67
≧65	25	9.92	14	18.67
Educational attainment				
None/Elementary	40	15.87	30	40.00
Junior high school	46	18.25	11	14.67
Senior high/vocational school	106	42.06	20	26.67
College/university	60	23.81	14	18.67
Marital status				
Married	189	75.00	53	70.67
Single	14	5.56	2	2.67
Divorced/separated/widowed	49	19.45	20	26.67
Monthly household income				
≦US$1,000	68	26.98	27	36.00
US$1,001–US$2,000	56	22.22	22	29.33
≧US$2,001	128	50.80	26	34.67
Living condition				
Living alone	19	7.54	6	8.00
Living with family or friends	233	92.46	69	92.00
Religion benefiting recovery				
No	31	12.30	21	28.00
Yes	221	87.70	54	72.00
Comorbidity				
None	142	56.35	43	57.33
Diabetes	25	9.92	5	6.67
Hypertension	66	26.19	16	21.33
Heart disease	26	10.32	6	8.00
Live disease	15	5.95	6	8.00
Asthma	3	1.19	2	2.67
Gout	5	1.98	2	2.67
Arthritis	23	9.13	8	10.67
Kidney disease	4	1.59	2	2.67
Dialysis	1	0.40	0	0.00
Other disease	13	5.16	2	2.67
Smoking habit				
Nonsmoker	243	96.43	64	85.33
Quit smoking	5	1.98	4	5.33
Occasionally	1	0.40	3	4.00
1–2 days/week	2	0.79	0	0.00
3–5 days/week	0	0.00	2	2.67
Most days	1	0.40	2	2.67
Alcohol drinking habit				
Nondrinker	221	87.70	64	85.33
Quit drinking	9	3.57	4	5.33
Occasionally	21	8.33	6	8.00
1–2 days/week	1	0.40	0	0.00
3–5 days/week	0	0.00	0	0.00
Most days	0	0.00	1	1.33
Exercise habit				
Never	30	11.90	11	14.67
Occasionally	45	17.86	26	34.67
1–2 days/week	29	11.51	10	13.33
3–5 days/week	77	30.56	14	18.67
Most days	71	28.17	14	18.67
Medical institution				
Regional hospital	43	17.06	20	26.67
Medical center	209	82.94	55	73.33
Cancer stage				
I	109	43.25	55	73.33
II	95	37.70	14	18.67
III	45	17.86	6	8.00
IV	3	1.19	0	0.00
Age at diagnosis (years)	Mean:48.89 ± 8.53		Mean:49.00 ± 10.30	
≤ 44	70	27.78	24	32.00
45–54	124	49.21	31	41.33
55–64	45	17.86	13	17.33
≥ 65	13	5.16	7	9.33
Treatment method				
Surgery	3	1.19	37	49.33
RT	1	0.40	3	4.00
Chemotherapy	5	1.98	1	1.33
Surgery & RT	2	0.79	9	12.00
Surgery & chemotherapy	25	9.92	6	8.00
RT & chemotherapy	2	0.79	10	13.33
Surgery & RT& chemotherapy	10	3.97	0	12.00
Surgery & HT	21	8.33	0	0.00
Surgery & RT & HT	24	9.52	0	0.00
Surgery & chemotherapy& HT	54	21.43	0	0.00
Surgery & RT& chemotherapy & HT	77	30.56	0	0.00
Surgery & RT & chemotherapy & targeted therapy	6	2.38	0	0.00
Surgery & RT & chemotherapy & HT & targeted therapy	15	5.95	0	0.00
other^a^	7	2.78	0	0.00
Illness duration (years)	Mean:5.58 ± 2.58		Mean:6.26 ± 4.19	
≧2 and < 3	32	12.70	15	20.00
≧3 and < 4	52	20.63	12	16.00
≧4 and < 5	44	17.46	14	18.67
≧5	124	49.21	34	45.33
Cancer recurrence or metastasization				
No	240	95.24	73	97.33
Yes	12	4.76	2	2.67

^a^Others includes: chemotherapy & HT, surgery & chemotherapy & targeted therapy, surgery & RT& HT & targeted therapy, and surgery & chemotherapy & HT & targeted therapy

### Health condition, health behavior and cancer treatment condition

Regarding the health condition of the breast cancer and cervical cancer survivors (Table 1), 56.35 and 57.33% did not have any comorbidities and 96.43 and 85.33% were nonsmokers, respectively. Regarding the severity of the patients’ cancer, 43.25 and 37.70% of the breast cancer survivors had stage I and II cancer, respectively, whereas most of the cervical cancer patients (73.33%) had stage I cancer. Most of the breast cancer survivors received a combination of surgical treatment, radiation therapy (RT), chemotherapy (CH) and HT (30.56%); followed by a combination of surgical treatment, CH and HT (21.43%); whereas most of the cervical cancer patients received surgical treatment alone (49.33%), followed by a combination of RT and CH (13.33%). The average illness duration among the breast and cervical cancer survivors at the time of recruitment was 5.58 ± 2.58 and 6.26 ± 4.19 years, respectively and 49.21% of the breast cancer survivors and 45.33% of the cervical cancer survivors had cancer for >5 years. The proportion of cancer recurrence and metastasis was 4.76 and 2.67%, respectively.

### Quality of life of the participants

First, the results of the QLQ-C30 (Table 2) indicated that the global health status of the two groups was similar. The mean score was 75.33 ± 20.25 (range, 8.33 ~ 100) for the breast cancer survivors and 75.56 ± 17.93 (range, 33.33 ~ 100) for the cervical cancer survivors. Regarding the functioning dimension, breast cancer survivors scored the lowest in the cognitive dimension (77.12 ± 19.42), whereas the cervical cancer survivors had the lowest scores in the emotional dimension (85.22 ± 18.00). In the symptom dimension, the three leading symptoms experienced by the breast cancer survivors were insomnia (26.06 ± 23.71), fatigue (19.27 ± 17.76), and constipation (15.08 ± 22.51), whereas those experienced by the cervical cancer survivors were insomnia (16.89 ± 24.73), constipation (16.89 ± 22.84), and fatigue (14.81 ± 16.87). The results of the EORTC QLQ revealed that the highest scores in the functioning dimension were for body image (breast cancer, 83.04 ± 22.38; cervical cancer, 89.93 ± 15.41), whereas the lowest scores were for sexual/vaginal functioning (breast cancer, 13.49 ± 16.66) and sexual activity (cervical cancer, 16.00 ± 22.83). In the symptom dimension, the most severe symptoms were hair loss (breast cancer, 23.56 ± 25.22) and peripheral neuropathy (cervical cancer, 16.89 ± 20.04).

**Table 2:** Core quality of life of the study participants

Variable	Breast cancer (*N* = 252)			Cervical cancer (*N* = 75)		
	Mean	SD	*P* ^a^	Mean	SD	*P* ^a^
Global health status (Overall QOL score) ^b^	75.33	20.25		75.56	17.93	
Age			0.823			0.935
≤ 44	73.55	18.06		75.00	14.91	
45–54	75.39	19.17		75.62	13.85	
55–64	74.83	21.58		77.17	22.50	
≥ 65	78.67	22.06		73.21	20.20	
Educational attainment			0.435			0.811
None/Elementary	75.63	25.76		77.22	15.15	
Junior high school	71.01	21.50		72.73	25.30	
Senior high/vocational school	76.02	18.71		73.33	18.06	
College/university	77.22	17.62		77.38	18.03	
Marital status			0.835			0.870
Married	75.40	20.38		76.26	18.45	
Single	77.98	18.95		75.00	35.36	
Divorced/separated/Widows	74.32	20.40		73.75	15.83	
Monthly household income			0.002			0.008
≦US$1,000	68.63	24.01		67.28	21.8	
US$1,001- US$2,000	74.26	16.54		78.41	11.97	
≧US$2,001	79.36	18.62		81.73	14.73	
Number of comorbidities			0.089			0.926
0	77.11	19.71		75.39	19.75	
1	75.82	18.24		74.21	15.57	
2	71.81	22.94		78.57	9.45	
≧3	64.44	24.08		79.17	25.00	
Medical institution			0.462^c^			0.936^c^
Regional hospital	73.26	23.26		75.83	23.40	
Medical Center	75.76	19.61		75.45	15.75	
Cancer stage			0.007			0.988
I	75.38	19.91		75.45	18.52	
II	72.11	21.29		76.19	19.02	
III	81.48	17.85		75.00	10.54	
IV	83.33	16.67		00.00	00.00	
Cancer recurrence or metastasization			0.364^c^			0.300^c^
No	75.59	20.20		75.91	17.71	
Yes	70.14	21.46		62.50	29.46	
Treatment			0.159			0.525
Surgery	00.00	00.00		76.35	19.69	
Chemotherapy	90.00	14.91		00.00	00.00	
Surgery& Chemotherapy	70.67	27.55		83.33	9.13	
Surgery& RT & Chemotherapy	67.50	24.36		69.44	17.68	
Surgery & HT	69.44	26.92		00.00	00.00	
RT/Chemotherapy	00.00	00.00		75.00	21.52	
Surgery & RT	00.00	00.00		68.52	16.02	
Surgery & RT& HT	80.56	16.79		00.00	00.00	
Surgery & Chemotherapy & HT	75.15	18.84		00.00	00.00	
RT& Chemotherapy	00.00	00.00		80.00	15.32	
Surgery & RT & Chemotherapy& HT	74.46	18.40		69.44	17.68	
Surgery & RT & Chemotherapy & Targeted	86.11	19.48		00.00	00.00	
Surgery & RT& Chemotherapy& HT& Targeted	83.33	10.91		00.00	00.00	
other	76.11	16.92		00.00	00.00	
Illness duration			0.001			0.647
≧2 and <3 years	69.01	22.72		79.44	14.73	
≧3 and <5 years	80.99	18.08		74.36	15.44	
≧5 years	72.58	20.27		74.75	20.97	
QOL dimension						
Functioning dimension^d^						
Physical functioning	91.19	10.98		92.27	9.68	
Role functioning	94.05	13.38		95.33	12.12	
Emotional functioning	84.82	19.13		85.22	18.00	
Cognitive functioning	77.12	19.42		85.56	17.18	
Social functioning	86.18	20.66		92.00	14.85	
Symptom dimension^e^						
Fatigue	19.27	17.76		14.81	16.87	
Nausea and vomiting	3.84	10.23		2.89	7.44	
Pain	11.57	17.90		8.44	15.59	
Difficulty breathing	8.20	16.13		7.11	15.78	
Insomnia	26.06	23.71		16.89	24.73	
Lack of appetite	6.22	16.86		3.56	10.36	
Constipation	15.08	22.51		16.89	22.84	
Diarrhea	5.82	13.69		10.22	17.31	
Financial difficulty	13.89	23.36		10.67	22.70	

^a^ *P* value of ANOVA

^b^Global health status (i.e. Overall QOL score): 0–100, high scores indicate high QOL

^c^ *P* value of t-test

^d^Functioning dimension: 0–100, high scores indicate high functioning level

^e^Symptom dimension: 0–100, high scores indicate severe symptoms

### Relevant factors influencing quality of life

Bivariate ANOVA was employed to explore the relevant factors influencing the global health status of the cancer survivors. Table 3 indicates that the primary factors influencing the global health status of the breast cancer survivors were their monthly household income, number of comorbidities, stage of cancer, method of cancer treatment and duration of illness. Relative to those earning monthly household incomes of < US$1,000, patients with a monthly household income of > US$2,000 or US$1,000–US$2,000 had a significantly higher global health status (10.99 ± 3.83 and 8.75 ± 3.37, respectively; both *p* < 0.05). Compared with the participants without comorbidities, the global health status of those with three comorbidities was significantly lower (−15.27 ± 4.94; *p* < 0.05). Furthermore, compared with the participants who had stage I cancer, the global health status of those who had stage II cancer was significantly lower (−5.08 ± 2.53; *p* < 0.05). Compared with the participants who received CH alone, those who received a combination of CH and surgical treatment exhibited a lower global health status. The breast cancer survivors who were ill for 3–5 years had a significantly higher global health status (9.54 ± 3.73; *p* < 0.05) than that of the participants who had been ill for 2–3 years.

**Table 3:** Relevant factors influencing the global health status of the breast and cervical cancer survivors according to the multiple regression analysis

Variable	Breast cancer (*N* = 252)			Cervical cancer (*N* = 75)		
	β	SE	*P*	β	SE	*P*
Intercept	83.8	7.04	<0.001	66.45	3.83	<0.001
Monthly household income						
≦US$1,000 (reference)						
US$1,001–US$2,000	8.75	3.37	0.010	11.72	4.91	0.017
≥ US$2,001	10.99	3.83	0.004	15.45	3.55	<0.001
Number of comorbidities						
0 (reference)						
1	−0.61	2.69	0.820			
2	−7.15	3.66	0.051			
≧3	−15.27	4.94	0.002			
Cancer stage						
Stage I (reference)						
Stage II	−5.08	2.53	0.045	−1.01	1.89	0.591
Stage III	3.90	4.42	0.378	8.54	4.64	0.066
Stage IV	6.27	12.86	0.626			
Cancer recurrence or metastasization						
no (reference)						
yes	−4.44	5.27	0.400	−6.62	4.49	0.140
Treatment methods						
Chemotherapy (reference)						
Surgery & chemotherapy	−18.20	4.08	<0.001			
Surgery & RT & chemotherapy	−24.46	5.00	<0.001			
Surgery & HT	−25.96	6.10	<0.001			
Surgery & RT & HT	−20.23	3.40	<0.001			
Surgery & chemotherapy & HT	−15.37	3.16	<0.001			
Surgery & RT & chemotherapy & HT	−22.48	4.22	<0.001			
Surgery & RT & chemotherapy & HT & target treatment	−16.49	3.89	<0.001			
Others	−20.07	5.81	<0.001			
Illness duration						
≧2 and <3 years (reference)						
≧3 and <5 years	9.54	3.73	0.011			
≥ 5 years	3.76	2.78	0.176			

Table 3 suggests that monthly household income was the only relevant factor influencing the global health status of the cervical cancer survivors, and those with monthly incomes of US$1,000–US$2,000 or > US$2,000 had a significantly higher global health status (11.72 ± 4.91 and 15.45 ± 3.55, respectively; both *p* < 0.05) compared with those with a monthly income of < US$1,000. Among the cervical cancer survivors, the stage of cancer and cancer recurrence or metastasis did not lead to significant differences (*p* > 0.05) in the global health status.

## Discussion

### Global health status of the patients with cancer

Scott et al. [21] revealed that the global health status for breast cancer was 61.8 ± 24.6. Because of the difference in cancer stage, the global health status for the breast cancer survivors in our study was higher (75.33 ± 20.25). Our results are similar to those reported by Fehlauer et al. [28], who performed a 12-year follow-up of breast cancer survivors and reported that their global health status was high. Compared with a study by Huang et al. [29], in which breast cancer patients who had received their diagnosis at least 9 months prior and received surgical treatment had a global health status of 56.3, the breast cancer survivors in the present study with an illness duration of >2 years had a higher global health status. In the symptom dimension, fatigue and insomnia were the more serious symptoms experienced by the breast cancer survivors in the present study. In addition, this group had low emotional functioning and cognitive functioning, which is similar to the finding of Mols et al. [30], who indicated that anxiety influenced the global health status of the breast cancer survivors in that study. Such an influence is due to HT causing menopausal symptoms, namely insomnia, depression, hot flashes, nausea, vomiting and cognitive dysfunction [31].

A study conducted by Dahiya et al. [32] in India reported that after treatment, the global health status of advanced cervical cancer patients (excluding critically ill patients) was 59.5 ± 10.9. Another study by Kumar et al. [33] excluded cervical cancer patients with stage IV and reported a global health status of 77.9 ± 7.17 for cervical cancer patients at 6 months after they had completed cancer treatment. In our study, the global health status for the cervical cancer survivors was 75.6 ± 17.9. The differences in the global health status scores between these studies is related to the differences in cancer stage, treatment methods and level of comorbidity [18].

Regarding the functioning dimension, the cervical cancer survivors in the present study had poor emotional functioning and typically experienced fatigue, insomnia, constipation and financial difficulty. Such findings are similar to those of Park et al. [16]. In contrast to the results of Hsu et al. [34], our results present more favorable scores in the patients’ global health status and the functioning and symptom dimensions. This difference might be because Hsu et al. focused on stage IB and IIA cervical cancer patients with an mean age was 61.5 ± 11.4 years, whereas the participants recruited in the present study were breast and cervical cancer survivors with mean ages of 54.48 ± 8.33 and 55.26 ± 10.50 years, respectively and who had stage IA cancer (22.7%). Compared with other related studies [16, 34, 35], the global health status of the breast cancer survivors (75.33 ± 20.25) and cervical cancer survivors (75.56 ± 17.93) in the present study indicate that they had a higher global health status, more favorable functional performance and experienced less severe symptoms. The higher scores might be because most of the participants recruited in the present study had early-stage cancer. For example, >60% of the breast cancer patients had stage I or stage IIA cancer, and a high proportion of cervical cancer survivors had stage I or II cancer.

### Relevant factors influencing the quality of life of cancer survivors

The factors influencing the QOL of breast cancer survivors were household income, number of comorbidities, stage of cancer, method of treatment and duration of illness. After the other factors were controlled for, the patients who received multitherapy (including surgical treatment) had a significantly lower global health status than those who received CH alone. Because patients with breast cancer typically receive a mastectomy or complete axillary lymph node dissection, they are likely to experience such symptoms as shoulder pain, swelling of the arms and inability to stretch the arms; moreover, such symptoms are generally persistent. Patients who receive a complete axillary lymph node dissection or RT are likely to experience allergy symptoms following surgery, and such conditions cause inconveniences in their daily life, thereby affecting their QOL. Previous studies have indicated that the physiological discomfort experienced by breast cancer survivors after treatment differs according to the type of treatment they received [36].

In order to reduce cancer survivors’ discomfort or recover their health, for the cancer survivors who have received multitherapy, they not only need to receive a long term plan of rehabilitation but also need to have psychological consultations.

High QOL was observed among patients with a high household income or who had been ill for a long period. These findings are similar to previous findings [37–39]. In the present study, the breast cancer survivors who had an illness duration of ≥3 years had higher QOL compared with those who had been ill for 2–3 years; among these patients, the breast cancer survivors with an illness duration of 3–5 years attained the highest QOL scores. Based on a short illness duration, we considered that the treatment method may influence the speed of physical recovery; patients who received multitherapy recovered more slowly. Although most of the participants in this study were long-term cancer survivors (>80% had an illness duration of ≥3 years), the influence of the cancer treatment methods on their current QOL was persistent. Therefore, this might explain why the cancer survivors’ current global health status was lower among those who underwent a combination of surgery and other treatment methods than among those who underwent CH alone. The other factors influencing the QOL of cancer survivors require further exploration.

Household income was the only relevant factor influencing the global health status of the cervical cancer survivors, with higher household incomes indicating a significantly higher global health status. Because only 75 cervical cancer survivors were recruited in this study, future studies should consider investigating other relevant factors influencing the global health status of cervical cancer survivors and increasing the sample size.

### Strengths and limitations

This study collected data on the global health status of cancer survivors with two of the most common cancers affecting women. The main strength of this study is that it not only simultaneously explored the associated factors of global health status for cancer survivors but also compared the differences in QOL and global health status between breast cancer and cervical cancer survivors. The limitations of this study are the lack of information on nonresponse cases. In addition, because we could not interview cancer survivors through random selection methods, the generalizability of the study results to all Taiwanese breast and cervical cancer survivors is limited. However, our study results may provide a reference for other countries.

## Conclusions

Regarding the QOL dimensions, the participants exhibited lower cognitive and emotional functioning and experienced more severe fatigue and insomnia. Compared with the cervical cancer survivors, the breast cancer survivors had lower QOL in all functioning dimensions. In addition, compared with the cervical cancer survivors, a higher proportion of breast cancer survivors were concerned about cancer recurrence and metastasis.

Household income, comorbidities, cancer stage, method of treatment and illness duration were relevant factors influencing the global health status of the breast cancer survivors; however, household income was the only factor influencing that of the cervical cancer survivors.

## Abbreviations

ANOVA: Analysis of variance
CVI: Content validity index
EORTC: European Organization of Research and Treatment for Cancer
HT: Hormone therapy
IRB: Institutional review board
OR: Odds ratio
QLQ-BR23: Quality of Life Questionnaire-Breast Cancer Module 23
QLQ-C30: Quality of Life Questionnaire-Core 30
QLQ-CX24: Quality of Life Questionnaire-Cervical Cancer Module 24
QOL: Quality of life
RT: Radiation therapy
